# New insights into the potential utility of the left atrial function analysis in heart failure with preserved ejection fraction diagnosis

**DOI:** 10.1371/journal.pone.0267962

**Published:** 2022-05-04

**Authors:** Roxana Cristina Rimbas, Ionela Simona Visoiu, Stefania Lucia Magda, Sorina Mihaila-Baldea, Maria Luiza Luchian, Alexandra Maria Chitroceanu, Memis Hayat, Diana Janina Mihalcea, Ruxandra Dragoi-Galrinho-Antunes-Guerra, Miruna Stefan, Andreea Velcea, Anca Andreea Andronic, Laura Lungeanu-Juravle, Alina Ioana Nicula, Dragos Vinereanu

**Affiliations:** 1 Cardiology and Cardiovascular Surgery Department, University of Medicine and Pharmacy Carol Davila, Bucharest, Romania; 2 Cardiology and Cardiovascular Surgery Department, University and Emergency Hospital, Bucharest, Romania; 3 Radiology Department, University and Emergency Hospital, Bucharest, Romania; The Open University, UNITED KINGDOM

## Abstract

**Aims:**

None of the conventional echocardiographic parameters alone predict increased NTproBNP level and symptoms, making diagnosis of heart failure with preserved ejection fraction (HFpEF) very difficult in some cases, in resting condition. We evaluated LA functions by 2D speckle tracking echocardiography (STE) on top of conventional parameters in HFpEF and preHF patients with diastolic dysfunction (DD), in order to establish the added value of the LA deformation parameters in the diagnosis of HFpEF.

**Methods:**

We prospectively enrolled 125 patients, 88 with HFpEF (68±9 yrs), and 37 asymptomatic with similar risk factors with DD (preHF) (61±8 yrs). We evaluated them by NTproBNP, conventional DD parameters, and STE. Global longitudinal strain (GS) was added. LA reservoir (R), conduit (C), and pump function (CT) were assessed both by volumetric and STE. 2 reservoir strain (S) derived indices were also measured, stiffness (SI) and distensibility index (DI).

**Results:**

LA R and CT functions were significantly reduced in HFpEF compared to preHF group (all p<0.001), whereas conduit was similarly in both groups. SI was increased, whereas DI was reduced in HFpEF group (p<0.001). By adding LA strain analysis, from all echocardiographic parameters, SR_CT<-1.66/s and DI<0.57 (AUC = 0.76, p<0.001) demonstrated the highest accuracy to identify HFpEF diagnosis. However, by multivariate logistic regression, the model that best identifies HFpEF included only SR_CT, GS and sPAP (R^2^ = 0.506, p<0.001). Moreover, SR_CT, DI, and sPAP registered significant correlation with NTproBNP level.

**Conclusions:**

By adding LA functional analysis, we might improve the HFpEF diagnosis accuracy, compared to present guidelines. LA pump function is the only one able to differentiates preHF from HFpEF patients at rest. A value of SR_CT < -1.66/s outperformed conventional parameters from the scoring system, reservoir strain, and LA overload indices in HFpEF diagnosis. We suggest that LA function by STE could be incorporated in the current protocol for HFpEF diagnosis at rest as a major functional criterion, in order to improve diagnostic algorithm, and also in the follow-up of patients with risk factors and DD, as a prognostic marker. Future studies are needed to validate our findings.

## Introduction

Heart failure (HF) with preserved ejection fraction (HFpEF) has a growing prevalence, accounting for approximately 50% of patients with clinical HF syndrome [1–3]. However, despite preserved left ventricular ejection fraction (LVEF), HFpEF patients have high morbidity and mortality, almost similar to patients with reduced EF (HFrEF) [4]. Because the pathophysiological mechanisms underlying HFpEF remain incompletely understood, there is no standard diagnostic algorithm [3–8], and little therapeutic progress has been achieved [9]. Although the newly proposed algorithms H2FPEF [5] and HFA-PEFF [6] tried to identify better HFpEF patients, a significant fraction of patients are still classified discordantly or are classified in the intermediate-likelihood category, where additional testing are needed [3, 6–8]. The current definitions have some limitations: the subjectivity of the symptoms, and the unfeasibility of measurements of cardiac output or filling pressure (invasive), being the most important [10]. Moreover, in real world scenario, not all patients are able to perform echocardiographic exercise stress tests, proposed by the HFpEF consensus. Elevated NTproBNP support, but normal levels do not exclude diagnosis of HFpEF, especially in treated and obese patients [6, 10].

Since 2D echocardiography (2DE) in resting conditions is the most important piece in the diagnosis of HFpEF, we need to better understand and define the transition from asymptomatic DD stage (pre-HFpEF) to symptomatic stage (HFpEF), in terms of echocardiographic parameters [1, 5, 6, 10, 11]. However, none of the conventional parameters alone predict increased NTproBNP level: E/A ratio, E’ velocities, E/E’ ratio, systolic pulmonary arterial pressure (sPAP), and left atrial maximal volume indexed (LAVi max) [12]. The present diagnosis of HFpEF depends on the level of NTproBNP and echocardiographic data, but the sensitivities of both are quite low [10, 13, 14]. In summary, we need an easy-to-do echocardiographic marker, or a sum of markers, able to predict HFpEF, regardless of NTproBNP level. These markers should be thought as a stamp of myocardial dysfunctionality that can be revealed by changing hemodynamic conditions.

In the earlier stage, LV filling pressure (LVFP) is normal at rest, but markedly increases during exercise, while in an advanced stage it increases continuously even at rest [15]. Consequently, prolonged increased LVFP generates structural and functional remodelling of the LA [14]. LAVi assessed by 2DE is already incorporated in new HFpEF scoring system [6], using well established cut-off values for grading severity. These values have strong evidence for prognosis in HF [16]. However, LAVi has no evidence in prediction of HFpEF diagnosis alone, and it is insufficient to identify LA dysfunction. LA deformation analysis, particularly LA reservoir strain, by STE appears to be robust to detect LA dysfunction, and has been shown to carry prognostic value in patients with HFpEF [17, 18].

Previous studies reported preclinical atrial dysfunction, characterized by reduced reservoir and conduit function, with preserved contractile function in stage A of American College of Cardiology/American Heart Association (ACC/AHA) HF stages, prior to overt LA enlargement [19, 20]. Progressive subclinical dysfunction from stage B ACC/AHA leads to a consistently reduction in reservoir and conduit function, with compensatory increase of pump function [20]. Clinical HF, stages C and D, occur when contractile function fails to compensate for reservoir and conduit dysfunction, advanced symptomatic HFpEF being characterized by a significant decrease in all 3 atrial functions [19, 21–23]. At present, there are no clear data regarding parameters that are able to diagnose HFpEF in patients with risk factors and symptoms. This is very important, since the essence of HF treatment is to prevent development of HF in patient with risk factors.

Therefore, we conducted this study to evaluate the added value of the LA functions by STE in diagnosis of HFpEF, on top of conventional parameters used in HFpEF diagnosis.

## Materials and methods

### Study population

We prospectively enrolled consecutive ambulatory patients with cardiovascular risk factors, signs and symptoms of HF, from January 2018 to January 2020. All patients were referred to our dedicated excellence HF centre to establish if they have HFpEF, according to guidelines [4]. In order to evaluate the prediction power of each echocardiographic parameter for HFpEF diagnosis, patients with HFpEF were compared to a group of patients with similar risk factor profile and DD, but without HF signs and symptoms, and normal NTproBNP level (preHF group), also prospectively enrolled on a 2:1 ratio model (HFpEF:preHF). The study protocol conforms to the ethical guidelines as reflected in a priori approval by the Institution’s Human Research Committee of the Carol Davila University of Medicine and Pharmacy. Informed consent was obtained in all subjects prior to enrolment.

Inclusion criteria for HFpEF group were: age > 18 years, sinus rhythm, stable patient with clinical, biological, and echocardiographic criteria suggestive for HFpEF, according to 2016 guidelines [4], informed consent signed. Exclusion criteria were recent hospitalization for acute HF (< 4weeks), sustained atrial/ventricular arrhythmia, significant valvular heart disease, hypertrophic cardiomyopathy, pericardial disease, previous history of myocarditis, any systemic inflammatory disease or vasculitis, active cancer in the last year, renal failure with haemodialysis, pulmonary causes of dyspnoea, moderate to severe anaemia, inappropriate quality of echocardiographic images for STE analysis. preHF group was selected from the asymptomatic patients with risk factors, without clinical and biological criteria for HFpEF. All patients had clinical examination, 12-lead electrocardiogram, screening laboratory tests, NTproBNP, and a comprehensive 2DE.

Demographic and clinical data were collected before echo protocol (age, gender, BMI, heart rate, systolic and diastolic blood pressure, HF symptoms and NYHA class, if any, HF aetiology, risk factors (hypertension, diabetes mellitus, smoker status, pulmonary diseases, sleep apnea, obesity, dyslipidaemia, ischemic disease documented by ECG, coronarography or other imaging technique). Treatment with all potentially cardiovascular active drugs was recorded on enrolment. After complete evaluation of the patients, the HFA-PEFF score was calculated, according to the current consensus [6].

### Echocardiographic protocol

**Conventional echocardiography** was performed using a Vivid E9 echocardiographic ultrasound system (GE Healthcare, Horten, Norway) with a 3.5 MHz transducer. The electrocardiographic tracing was adjusted to show a well-defined P wave. Standard images were acquired and digitally stored for offline analysis using a vendor specific software Echo PAC PC, version BT12. Echocardiographic protocol included three apical views (four-chamber, two-chamber, and long-axis) optimized for LV, followed by dedicated apical four-chamber and two-chamber views for LA, avoiding foreshortening of the LV and LA, during acquisition. For each view, three consecutive heart cycles were recorded with a frame rate ranging between 50 and 80 frames/sec. A good quality electrocardiogram (ECG) trace with well visible P was recorded. All images were digitally stored and exported to a separate workstation for offline analysis. The operators performing the echocardiographic analysis were blinded to the patient’s clinical and lab details. The quantification of the cardiac chamber size and function was performed in agreement with the current guidelines [16, 24]. LV mass indexed (LVMI) was calculated using the linear method from the parasternal long axis view (Cube formula) and indexed to the body surface area (BSA) [16].

LAVi max was calculated using the biplane disk summation technique and indexed to BSA, as a mean from the apical 4- and 2-chamber views [16]. Transmitral pulsed-wave Doppler velocities (E, A) and tissue Doppler velocities of the septal and lateral mitral annulus were recorded and mean of both velocities was calculated (E’). Tricuspid regurgitant jet velocity and inferior vena cava diameter were measured for the estimation of the sPAP. DD was assessed in a step-by-step algorithm, based on the 2016 American Society of Echocardiography recommendations [24].

**LV global longitudinal strain (GS)** was measured from apical 2D views (four-, two-, and three-chamber views), by manually tracing the endocardial border of the LV at the end of systole, at the smallest LV chamber size. GS was calculated as the average strain values of all 18 LV segments, according to the guidelines [16].

#### LA volumetric and functional assessment by 2DE

LA analysis was performed by two experienced operators, experts in STE analysis (RCR and SMB). LA volumetric assessment was done from 4C and 2C dedicated views, and reported as a mean value and indexed by BSA [25], as follows:

- LA maximal volume (LAVi max)—volume at the LV end-systole, before the mitral valve opening.
- LA pre-A volume (LAVi pre-A)—volume before the onset of the P-wave on the ECG tracing.
- LA minimal volume (LAVi min)—volume at the LV end-diastole, after mitral valve closure.

**LA phasic functions** were generated by using published formulas, based on LA volumes [25]:

- **Reservoir function**:
$$\text{LA}\;\text{total}\;\text{emptying}\;\text{fraction}\;\left(\text{EF}\right)=\left(\text{LAV}\;\text{max}–\text{LAV}\;\text{min}\right)/\text{LAV}\;\text{max}\;\text{x}\;100$$
$$\text{LA}\;\text{expansion}\;\text{index}=\left(\text{LAV}\;\text{max}–\text{LAV}\;\text{min}\right)/\text{LAV}\;\text{min}\;\text{x}\;100$$
- **Conduit function**: LA passive EF = (LAV max—LAV preA)/LAV max x 100
- **Pump function**: LA active EF = (LAV preA–LAV min)/LAV preA x100

#### LA longitudinal deformation analysis by STE

**Fig 1:**
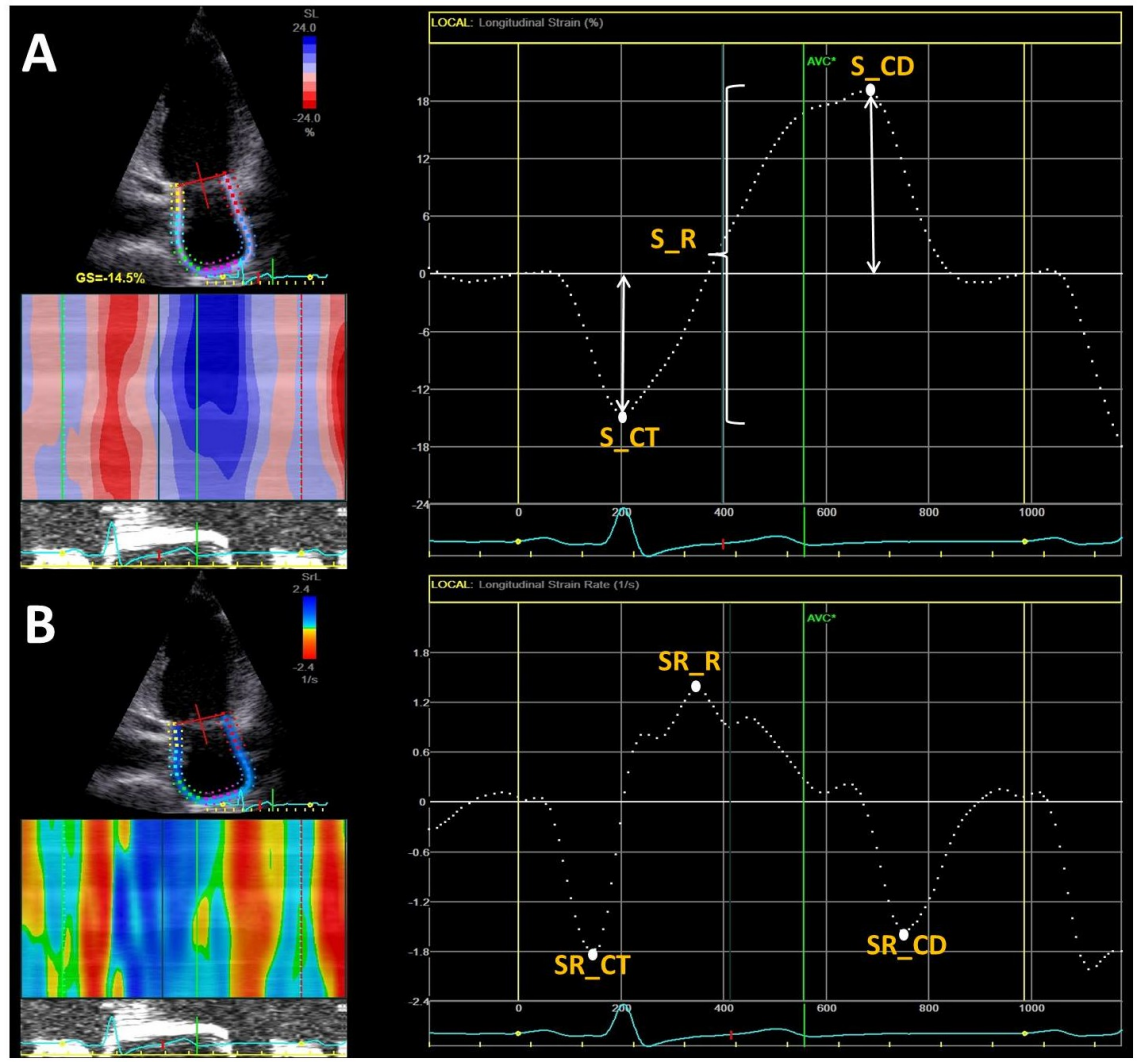
LA strain by speckle tracking echocardiography. Example of left atrial strain (panel A) and strain rate (panel B) from four-chamber apical view, using speckle tracking echocardiography, illustrating reservoir, conduit and pump deformation in a diastolic dysfunction patient. S_R, reservoir strain; SR_R, reservoir strain rate; S_CD, conduit strain; SR_CD, conduit strain rate; S_CT, contractile strain, SR_CT, contractile strain rate.

A detailed LA longitudinal deformation evaluation by 2DSTE was published previously by our echocardiography lab [26, 27]. In summary, the LA strain curves were generated by manually tracing the endocardial border in the apical four- and two-chamber views (P-P gating) [27, 28]. The average deformation values from the apical four- and two-chamber views were used for analysis. LA phasic functions were defined as follows:

- **LA reservoir function**: LA reservoir strain (S_R) deformation between MVC to MVO (calculated as the sum between the peak negative longitudinal strain (absolute value), and LA reservoir strain rate (SR_R) during systole, defined as peak positive strain rate from de strain rate curve (Fig 1).
- **LA conduit function**: LA conduit strain (S_CD) as peak positive strain (Fig 1A), and LA conduit strain rate (SR_CD) as peak negative strain rate during early LV diastole.
- **LA booster pump function**: LA contractile strain (S_CT) as peak negative strain (Fig 1A) and strain rate during late diastole, corresponding to atrial contraction, defined as and strain rate (SR_CT) (Fig 1B).
- **LA overload indices**. We defined two non-invasive indices as markers of LA overload:
  - **stiffness index (SI)**, estimated by the ratio between E/E’ ratio and S_R [29], as a marker of pressure overload, representing the amount of pressure required to induce a change in LA deformation during the reservoir phase.
  - **distensibility index (DI)**, estimated by the ratio between S_R and LAVi max, as a marker of volume overload, representing the amount of deformation for any volume change of LA, knowing that the excursion is lower for an already dilated LA [30]. We also evaluated the time added for measurement of all LA parameters, in order to estimate how much time adds a functional assessment of the LA at the conventional echocardiographic evaluation proposed by the guidelines.

### Reproducibility

Intra- and inter-observer variability from our echo lab for all conventional echocardiographic and TDI parameters, and also for STE parameters were already reported elsewhere [27, 31–33]. We also assessed intra- and inter-observer variability for all LA strain and strain rate parameters, on 10 patients that were randomly selected, using an online random selection generator. The intra-observer assessment was performed at 2 weeks apart (RRC). For inter-observer variability assessment, the same patients were analysed by a second blinded observer (SMB).

### Statistical analysis

All continuous variables were assessed for the normal distribution by Kolmogorov–Smirnov test. Normally distributed continuous variables were reported as mean ± SD and compared for statistical significance with Independent Samples T Test. Non-normally distributed continuous variables were presented as the median and interquartile range (IQR) and compared using the Mann Whitney *U* Test. A p value of <0.05 was considered significant. Categorical variables were expressed as percentages and compared with Chi-square test. Correlation between continuous variables was performed using Pearson’s or Spearman’s correlation coefficient as appropriate. Multivariate logistic regression was used to identify predictors of HFpEF and to calculate the corresponding odds ratios. The receiver operating characteristic (ROC) curve was used to identify prediction of HFpEF diagnosis for each echocardiographic parameter and to determine cut-off values. Sensitivity (Se) and specificity (Sp) were calculated. ICCs and their 95% confident intervals were calculated usingSPSS statistical package, based on an absolute-agreement, 2-way mixed-effects model. Statistical analysis was performed using SPSS version 21 (SPSS Inc., Chicago, IL, USA).

## Results

### Demographics and clinical data

We screened 100 symptomatic patients potentially having HFpEF, and 50 asymptomatic patients with cardiovascular risk factors and DD (preHF). We enrolled prospectively 125 patients, 88 patients with stable HFpEF (HFpEF group), and 37 asymptomatic patients, with cardiovascular risk factors and LVDD (preHF group). 12 patients were excluded from HFpEF group (2 with severe pulmonary diseases, 2 hypertrophic cardiomyopathy, 3 severe anaemia, 5 with images unsuitable for STE analysis). Patients excluded based on the echocardiographic evaluation were because of the inability to obtain optimal images, either for LV or for LA analysis, an essential part of the STE evaluation.

We excluded 13 patients from asymptomatic patients with RF because they do not have DD, according to 2016 guideline. Patients’ characteristics are summarized in Table 1. The two groups are almost similar in terms of demographic and clinical data, except that the HFpEF patients are older (Table 1). As expected, NT-proBNP level was significantly higher in HFpEF group, median 197 pg/ml, IQR (143, 318) vs preHF group, median 42 pg/ml, IQR (24, 68) (P<0.001).

**Table 1:** Demographic and clinical characteristics of all patients.

Parameter	preHFpEF	HFpEF	*P* value
	(n = 37)	(n = 88)	
Age (yrs)	61.4±8.3	67.5±9.1	0.001
Female (%)	62	75	0.15
BMI (kg/m^2^)	30.9±4.5	29.7±4.9	0.2
SBP (mmHg)	142±17	141±22	0.91
DBP (mmHg)	85±9	80±11	0.03
HR (bpm)	69±9	69±10	0.9
Smoker (%)	24	14.8	0.1
Arterial hypertension (%)	97.	93.	0.3
Dyslipidaemia (%)	89	89	0.8
Diabetes mellitus (%)	46	31	0.12
AFib (n, %)	0	17 (19%)	<0.001
BMI >25kg/m^2^ (%)	54	49	0.5
HFA-PEFF score	-	5.8±0.6	
NYHA class			
Class II (%)	-	96.4	
Class III (%)	-	3.6	

AFib, atrial fibrillation in the medical history. BMI-body mass index, SBP-systolic blood pressure, DBP-diastolic blood pressure, HR-heart rate.

### LV structure and functions

LV dimensions, volumes, LVMI, and LVEF were similar in HFpEF and DD groups, but GS was impaired in HFpEF as we expected (Table 2). HFpEF group displayed lower mean E’ velocity, higher E/E’ ratio and sPAP. Regarding the severity of DD, grade 1 predominated in preHF group, whereas grade 2 in HFpEF group (Table 2).

**Table 2:** Comparison between groups in terms of systolic and diastolic parameters.

Parameter	preHFpEF	HFpEF	*P* value
	(n = 37)	(n = 88)	
**LV structure and systolic function**			
IVS (mm)	12.4±1.9	12.0±2.0	0.43
PW (mm)	11±1.8	10.9±1.7	0.95
LVEDD (mm)	43.6±4.6	45.3±5.3	0.09
LVEDVi (ml/m^2^)	47.5±9.9	46.0±9.8	0.45
LVESVi (ml/m^2^)	19.97±6.9	18.74±5.5	0.29
LVEF (%)	58.8±6.9	60.3±5.7	0.22
LVMI (kg/ m^2^)	94.6±19.6	103.2±26.3	0.08
GS (%)	-20±3	-18±3	**<0.001**
**Diastolic dysfunction**			
E (cm/s)	74.±16.	81.±22	0.07
A (cm/s)	88.±17.8	90.±22	0.48
E/A median (IQ)	0.82 (0.73, 099)	0.86 (0.71, 1.1)	0.37
E’ mean (cm/s)	8.3±1.6	7.5±1.7	**0.014**
E/E’ median (IQ)	9 (7.6, 10.6))	10.4 (8.8, 13)-	**0.03**
sPAP (mmHg)	27.9±7.6	34.6±8.5	**<0.001**
Grade I % (n)	81(30)	46 (40)	**<0.001**
Grade II % (n)	19 (7)	53 (48)	**<0.001**
Grade III % (n)	0 (0)	1(1)	0.52

E/A, ratio between peak early to late diastolic mitral inflow peak velocities; E/E’, ratio between early diastolic mitral inflow to mitral annular early diastolic tissue velocities; GS, global strain; IVS, interventricular septum; LVEDD, left ventricular end-diastolic diameter; LVEDVi, left ventricular end-diastolic volume indexed; LVESVi, left ventricular end-systolic volume indexed; LVEF, left ventricular ejection fraction; LVMI, left ventricular mass indexed, PW, posterior wall; sPAP, systolic pulmonary artery pressure.

### LA volumes and phasic functions

LAVi max, preA, and min were significantly higher in HFpEF by comparison to preHF group (p<0.001). Reservoir function was significantly impaired in HFpEF compared to preHF (Table 3). LA conduit function was similar in both groups. By contrast, LA pump function was significantly reduced in HFpEF, with lower values of the S_CT, and SR_CT (Table 3). SI was significantly higher, whereas DI was significantly lower in HFpEF compared to preHF group (p<0.001) (Table 3). Time added for all LA strain derived parameters analysis was 148±14 sec (138–159).

**Table 3:** Left atrial volumes and functions.

Parameter	preHFpEF	HFpEF	*P* value
	(n = 37)	(n = 88)	
**LA volumes**			
LAVi max (ml/m^2^)	40.3±9.2	48.6±11	**<0.001**
LAVi pre A(ml/m^2^)	27.8±6.9	34.3±10.2	**<0.001**
LAVi min (ml/m^2^)	16.7±5.2	22.5±8.8	**<0.001**
**LA functions**			
**Reservoir**			
Total EF (%)	58.7±8.0	54.6±9.1	**0.018**
Expansion index	151.6±51.4	128.6±43.2	**0.011**
S_R (%)	25.4±3.8	23.3±4.5	**0.014**
SR_R (1/s)	1.2±0.2	1.1±0.3	0.383
**Conduit**			
Passive EF (%)	31.0±8.9	30.0±7.1	0.508
S_CD (%)	10.9±3.2	10.1±3.3	0.172
SR_CD (1/s)	-1.15±0.4	-1.14±0.6	0.896
**Pump**			
Active EF (%)	40.1±8.5	35.1±9.7	**0.009**
S_CT (%)	-14.5±2.4	-13.2±3.1	**0.030**
SR_CT (1/s)	-1.8±0.4	-1.4±0.5	**<0.001**
**LA overload indexes**			
Stiffness index	0.4±0.1	0.5±0.2	**<0.001**
Distensibility index	0.7±0.2	0.5±0.2	**<0.001**

EF, emptying fraction; LA, left atrium, LAVi, left atrium volume indexed, S_R, reservoir strain. SR_R, reservoir strain rate; S_CD, conduit strain; SR_CD, conduit strain rate; S_CT, strain contraction; SR_CT, strain rate contraction.

### Correlations between NT-proBNP level and echocardiographic parameters

All parameters correlated to NT-proBNP are reported in Table 4. NTproBNP best correlated with sPAP, DI, and SR_CT (all p<0.001) (Table 4).

**Table 4:** Correlations between NT-proBNP and echo parameters.

Parameter	R correlation	*P* value
sPAP (mmHg)	0.422	<0.001
Distensibility index	-0.410	<0.001
SR_CT (1/s)	0.406	<0.001
LAVi min (ml/m^2^)	0.375	<0.001
LAVi max (ml/m^2^)	0.358	<0.001
Stiffness index	0.269	0.003
GS (%)	-0.262	0.002
E/E’	0.230	0.010
S_R (%)	-0.178	0.047

E/E’, ratio between early diastolic mitral inflow to mitral annular early diastolic tissue velocities; GS, global strain; LAVi, left atrium volume indexed; sPAP, systolic pulmonary artery pressure; S_R, reservoir strain; SR_CT, contractile strain rate.

### LA pump function correlations

**Fig 2:**
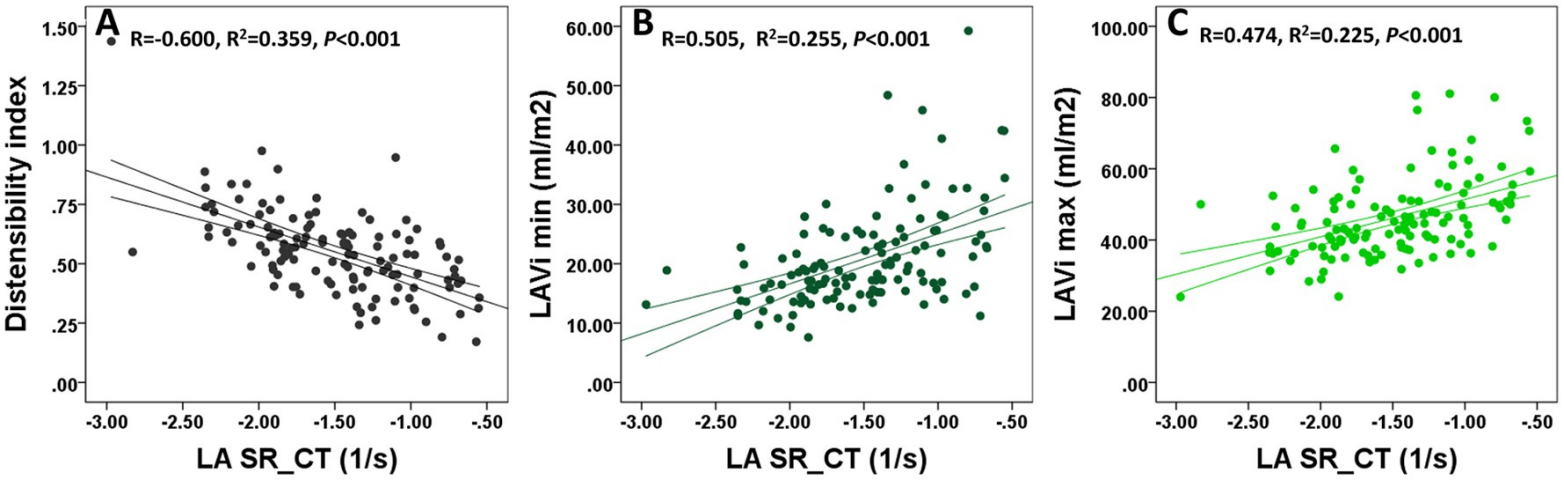
Correlations between LA pump function and other parameters. Correlations between LA pump function and distensibility index (Panel A), LAVi min (Panel B) and LAVi max (Panel C). LAVi, left atrium volume indexed; SR_CT, contractile strain rate.

Since from all LA deformation parameters the higher correlation of NT-proBNP was with SR_CT, we evaluated separately the additional correlations of this parameter. The best correlations were with DI, LAVi min and max, and S_CT (Fig 2). LAVi min correlated with SR_CT better then LAVi max. E/E’ ratio registered a weak correlation with SR_CT (Table 5).

**Table 5:** Correlations between LA pump function and other echo parameters.

Parameter	R correlation	*P* value
Distensibility index	-0.60	<0.001
LAVi min	0.51	<0.001
LAVi max	0.47	<0.001
S_R	-0.41	<0.001
Stiffness index	0.32	<0.001
GS	0.27	0.002
E/E’	0.18	0.048

E/E’, ratio between early diastolic mitral inflow to mitral annular early diastolic tissue velocities; GS, global strain; LAVi, left atrium volume indexed, S_R, reservoir strain.

### Diagnostic performance of the conventional and STE parameters for HFpEF diagnosis

**Fig 3:**
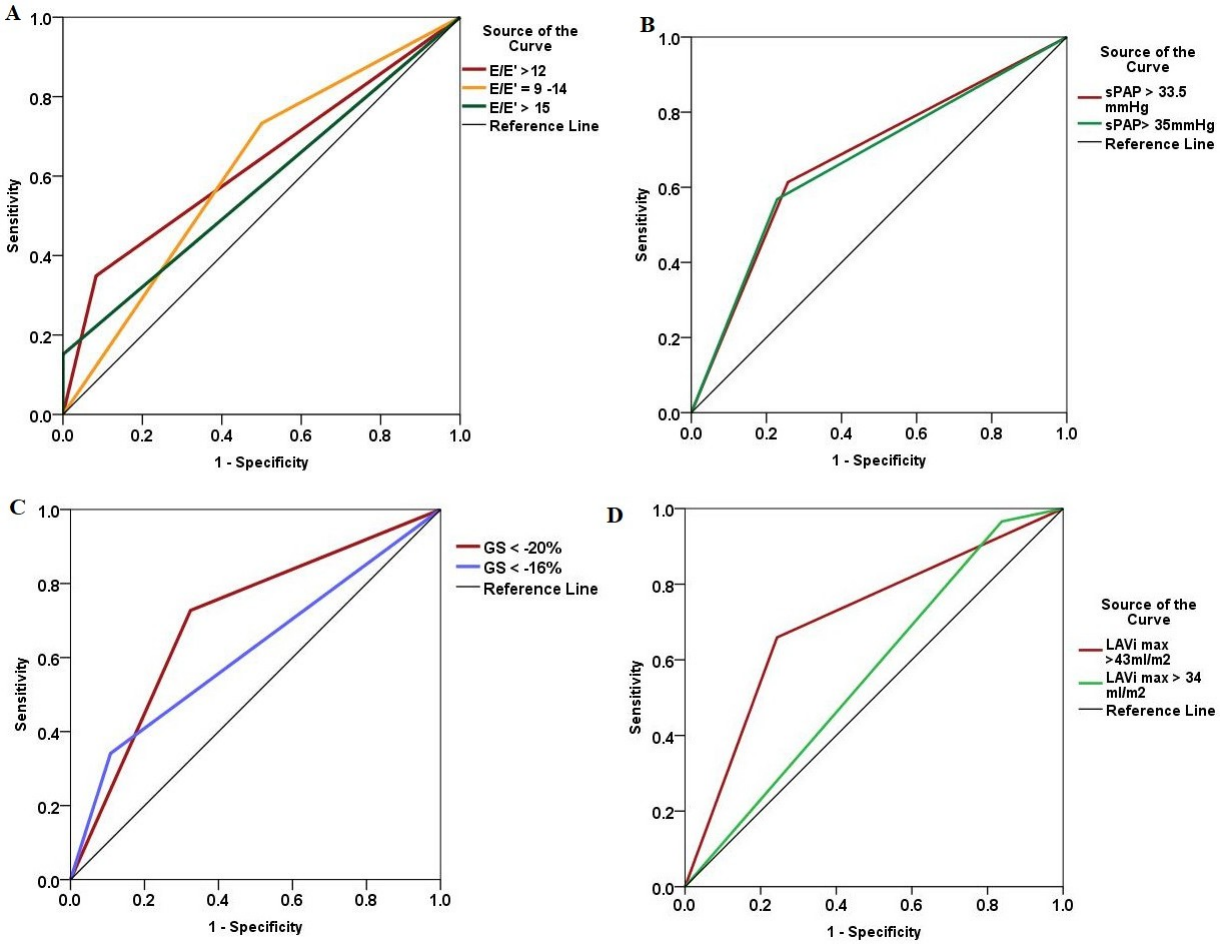
Comparative receiver-operating curves of the conventional echocardiographic parameters for HFpEF diagnosis, between new-proposed and old cut-off values. **Panel A**. E/E’ ratio >12 registered a higher accuracy (AUC = 0.633), compared to the old values 9–14 (AUC = 0.616) and >15 (AUC = 0.576); **Panel B**. Systolic pulmonary artery pressure (sPAP) > 33.5 mmHg (AUC = 0.678) showed a slightly higher accuracy compared to the guidelines value > 35 mmHg (AUC = 0.670). **Panel C**. Global strain (GS) < -20% demonstrated a significantly higher AUC = 0.727, compared to the consensus proposed value of < -16%; **Panel D**. Left atrial maximal volume indexed (LAVimax) > 43 ml/m^2^ predicts better patients with HFpEF, by comparison to the conventional cut-off value >34 ml/m^2^.

From all parameters included in the current HFpEF algorithm (6), with their cut-off values specified in the guidelines, sPAP >35 mmHg demonstrated the highest accuracy for HFpEF diagnosis, although with limited sensibility and specificity (Table 6, Fig 3). Parameters such as E/E’, E’ velocities, GS, LAVi max, LVMi, LVWT had low accuracy in detection of HFpEF (Table 6, Fig 3).

**Table 6:** Diagnostic accuracy for HFpEF diagnosis of the guideline and new suggested echocardiographic parameters.

	Cut-off values	AUC	p value	Sensitivity	Specificity
**LAVi max (ml/m** ^**2**^ **)**	>34	0.56 [0.45–0.679]	0.26	96.7%	16.2%
	**> 43**	**0.71 [0.6091–0.807]**	**<0.001**	**65.9%**	**75.7%**
**sPAP (mmHg)**	**>33.5**	**0.678 [0.575–0.782]**	**0.002**	**61%**	**74.3%**
	>35	0.670 [0.567–0.773]	0.003	56.8%	77.1
**E/E’**	**>12**	**0.633 [0.531–0.734]**	**0.02**	**34.9%**	**91.7%**
	9–14	0.616 [0.504–0.728]	0.04	73%	50%
	>15	0.576 [0.47–0.68]	0.19	15%	100%
**E’ medial/lateral (cm/s)**	>7/10	0.611 [0.501–0.68]	0.05	68.2%	54.1%
**LVWT (mm)**	>12	0.406 [0.299–0.51]	0.10	56.8%	24.3%
**LVMi (g/m** ^**2**^ **) (m/w)**	149/122	0.55 [0.443–0.65]	0.39	12%	97.3%
	115/95	0.56 [0.446–0.67]	0.33	52%	60%
**GS (%)**	**< -20**	**0.701 [0.611–0.810]**	**<0.001**	**72.7%**	**67.6%**
	< -16	0.616 [0.611–0.810]	0.04	66%	90%
**S_R (%)**	**< 25**	**0.635 [0.521–0.739]**	**0.017**	**69.3%**	**56.8%**
**S_CT (%)**	**> -14.7**	**0.627 [0.523–0.732]**	**0.025**	**71.6%**	**51.4%**
**SR_CT (1/s)**	**< -1.66**	**0.760 [0.608–0.811]**	**<0.001**	**71.6%**	**70.3%**
**Stiffness index**	**> 0.51**	**0.700 [0.609–0.792]**	**<0.001**	**37.5%**	**97.2%**
**Distensibility index**	**< 0.57**	**0.766 [0.685–0.866]**	**<0.001**	**69.3%**	**83.8%**

E’, diastolic tissue Doppler velocities; E/E’, ratio between early diastolic mitral inflow to mitral annular early diastolic tissue velocities; GS, global strain; LAVi, left atrium volume indexed; LVMi, left ventricle mass indexed; LVWT, left ventricle wall thickness, sPAP, systolic pulmonary artery pressure; S_R, reservoir strain; S_CT, contractile strain; SR_CT, contractile strain rate.

However, we identified a new cut-off value for LAVi max > 43 ml/m^2^, able to detect HFpEF diagnosis with higher specificity by comparison to the conventional cut-off value (>34 ml/m^2^) (Sp = 75.7% vs 16.2%) (p<0.001) (Table 6, Fig 3). Moreover, LAVi min > 19.8 ml/m^2^ had a higher specificity for HFpEF diagnosis than LAVi max. Although E/E’ >12 had very good Sp = 91.7%, the sensitivity for HFpEF diagnosis was extremely low (Table 6). However, by comparison with the cut-off values from the HF consensus, the accuracy was higher (Table 6). GS <-20%, was identified as having a higher AUC than GS < -16%, considered as minor functional criteria in the consensus (Table 6, Fig 3).

**Fig 4:**
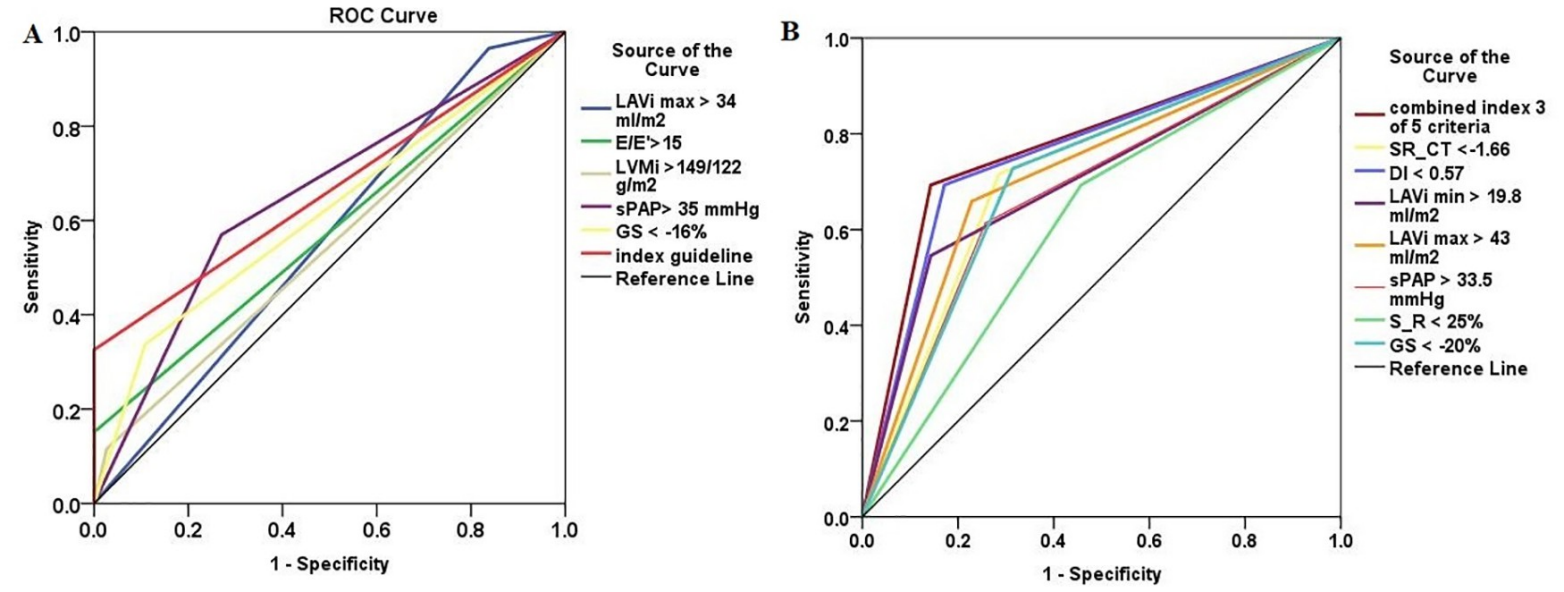
Comparative receiver-operating curves of guideline and new echocardiographic parameters for HFpEF diagnosis. The new echocardiographic parameters (**Panel B**) showed higher AUC compared to all current guideline parameters (**Panel A**). A combined index of 3 from 5 parameters with the highest AUC (SR_CT, sPAP, GS, DI, LAVi min) identified HFpEF better than the actual guideline index and all parameters alone, including the new ones. LAVi, left atrium volume indexed; E/E’, ratio between early diastolic mitral inflow to mitral annular early diastolic tissue velocities; LVMi, left ventricle mass indexed; sPAP, systolic pulmonary arterial pressure; GS, global strain; SR_CT, LA contractile strain rate; DI, distensibility index; S_R, LA reservoir strain.

From all strain derived parameters, DI and SR_CT were the best parameters able to identify HFpEF, with a cut-off value of <0.57 and < -1.66 /s, respectively (p<0.001). S_R<25% demonstrated lower accuracy for HFpEF diagnosis, by comparison with these two parameters (Fig 4, Table 6).

When compared the AUC for all newly defined cut-off values, DI and SR_CT were superior to all echocardiographic parameters used for diagnosing HFpEF in scoring system (Fig 4).

According to multivariate logistic regression, using all parameters defined in Table 6, the model that best identified HFpEF included only SR_CT < -1.66 **1/s**, sPAP >33.5 mmHg, and GS< - 20% (R^2^ = 0.506, p<0,001) (Table 7). Neither LAVi max/min, nor DI or SI were independent predictors. SR_CT < -1.66 1/s was an independent predictor of HFpEF, and had the most important role in this model, having the highest OR (Table 7).

**Table 7:** Predictors of HFpEF in multivariate logistic regression.

Parameter	OR [95% CI]	*P* value
**SR_CT**	**5.829 [1.389–24.451]**	**0.016**
GS	1.303 [1.066–1.592]	0.010
sPAP	1.129 [1.055–1.209]	0.001

GS, global strain; sPAP, systolic pulmonary arterial pressure; SR_CT, contractile strain rate.

Using parameters with the highest AUC, such as SR_CT, sPAP, GS, DI, LAVi min, with their cutt-off values, we create a combined model (1point for each parameters), in order to see if a combination of parameters might identify HFpEF better than conventional and new parameters alone. A combined index of 3 from 5 parameters identified better HFpEF than SR_CT, DI, sPAP, GS, and than guidelines combined index (AUC = 0.775, Se = 70, Sp = 86%, p<0.001) (Fig 4). By using 4 or 5 parameters of 5, the AUC (0.73 vs 0.62) and Se (52 vs 24%) decrease, whereas Sp increases (95% vs 100%), respectively.

Intra- and inter-observer reproducibility were good to excelent for all LA strain and strain rate parameters (Table 8).

**Table 8:** Reproducibility of the LA strain parameters by STE.

	Intraobserver			Interobserver		
Parameters	MN±SD	ICC	95% CI	MN±SD	ICC	95% CI
S_R	22.12±0.56	0.984	0.845–0.997	22.32±0.15	0.934	0.760–0.983
SR_R	1.09±0.03	0.976	0.891–0.994	1.09±0.03	0.800	0.391–0.946
S_CD (%)	9.38±0.29	0.981	0.924–0.995	9.60±0.14	0.937	0.771–0.984
SR_CD (1/s)	-0.9±0.02	0.974	0.902–0.993	-0.92±0.02	0.883	0.525–0.971
S_CT (%)	-12.73±0.27	0.987	0.885–0.997	-12,72±0.29	0.961	0.857–0.99
SR_CT (1/s)	-1.59±0.03	0.980	0.927–0.995	-1.612±0.02	0.970	0.891–0.992

MN, mean; SD, standard deviation; ICC, intraclass correlation coeficient; CI, confidence interval; S_R, reservoir strain; SR_R, reservoir strain rate; S_CD, conduit strain; SR_CD, conduit strain rate; S_CT, strain contraction; SR_CT, strain rate contraction.

## Discussions

In our prospective study we enrolled 125 patients, 88 with stable HFpEF, and 37 asymptomatic LVDD patients without HF, analysed by conventional 2DE and STE, to establish the added value of the LA function in the diagnosis of HFpEF, on top of conventional parameters from the scoring system.

### LA functional analysis

To the best of our knowledge, this is the first study that showed that LA pump function and distensibility index, evaluated by STE, are able to detect better HFpEF than any other structural or functional parameter from the 2019 scoring system. A value of SR_CT < -1.66/s and DI <0.57 outperformed conventional parameters, reservoir strain, and GS in HFpEF diagnosis. However, when clinical parameters, standard echocardiographic parameters, LA strain parameters, and GS were included in a multivariate logistic regression, the model that best predicted HFpEF, included only SR_CT, GS, and sPAP, SR_CT having the highest OR in this model. Our study also confirms significant impairment of the reservoir function, with similar conduit function in HFpEF compared to preHF group. Of note all LA strain and strain rate parameters had very good reproducibility in our study, similar to the most recent publications [33, 34].

By comparison to our study, previous studies have shown contradictory data on potential predictors of HFpEF. However, they compared patients with cardiac vs. non-cardiac dyspnoea, regardless of the presence or absence of DD, with other possible confounding factors in the control group [35–37]. Sanchis et al. [35] showed that DI is the best predictor for HF diagnosis. These results could be explained by different patients’ profile (older patients, severely dilated LA, mixed HFpEF and HFrEF patients). It worth mention that we found an almost similar cut-off value for SR_CT as Sanchis et al. (< -1.69/s), however with slightly lower sensitivity and specificity in our case. This might be explained by the differences in patient’s selection. Sanchis et al., in another paper, found that SR_CT< -1.40/s was the best parameter to evaluate prognosis in HF [38].

On the contrary, Reddy et al. [36], found that S_R outperformed the conventional used DD parameters. They found that S_R<24.5% can identify HFpEF (Se = 56%, Sp = 94%) very close to that identified by us, S_R < 25%. Moreover, they found that LA stiffness index was superior to S_R alone in diagnosis of HFpEF. Similarly, we found that SI was superior to S_R, when analysed alone, but both were inferior to SR_CT. The lack of discriminatory power of SR_CT in Reddy’s study might be related to the inclusion of patients with atrial fibrillation.

Compared to these two studies, in which in the control group GS was reduced, in our study, preHF patients had preserved GS, despite increased LAVi max and impaired LA phasic function. In this light, our findings confirm the hypothesis of an earlier LA failure in preHF patients [19].

Telles et al. demonstrated that HFpEF patients compared to patients with non-cardiac dyspnea (NCD), have decreased S_R (24.3±9.6 vs. 36.7±8.4%), SR_R (0.9±0.3 vs. 1.2±0.3/s), S_CT (-11.5±3.2 vs.-17.0±3.4%) and SR_CT (-1.1±0.5 vs. -1.6±0.4/s, p<0.001), with similar conduit function [37]. Our data confirm similar values for reservoir and pump strain in HFpEF patients, but different values for preHF group. Differences can be explained by the enrolment of patients with NCD as control group, without DD. Moreover, they also showed that S_CT identified HFpEF (Se = 94%, Sp = 80%, AUC = 0.88), while S_R (Se = 83%, Sp = 80%, AUC = 0.83) (P<0.001). Both reservoir and pump strain far exceeded the predictive ability of E/E’. By contrast, we found that SR_CT was superior to S_CT in prediction of HFpEF, because our HFpEF group had less severe symptoms (NYHA II class) and less severe dilated LA. The non-invasive SI was also higher in HFpEF patients, as in our study.

In another study, LVH magnitude and atrial dilation/failure provided the best separation between preHF and HFpEF group in hypertensive patients. Their results suggested that atrial function may be a key compensatory mechanism countering evolution of HFpEF, and they highlight the diagnostic utility of atrial failure as a marker of the disease, too [39].

### LA morphological analysis

In our study, all LA volumes were significantly increased in HFpEF patients compared to preHF patients. LAVimax >43 ml/m^2^ can predict HFpEF with significant increased specificity when compared to the guideline value (>34 ml/m^2^). However, when LAVimax was included in the regression analysis, it did not predict HFpEF. Moreover, we found a better correlation of LAVi min, with SR_CT compared to LAVimax. Previous studies suggested that LAVimin, is a marker of atrial afterload and is more closely related to LVFP, being strongly associated with NT-proBNP than LAVimax [40–42]. Russo et al. [40] found that LAVimin had a strong association with E/E’ ratio, whereas LAVimax had not. Recently, Thadani et al. [42] demonstrated a better predictive power for LAVimin compared to LAVimax for HF hospitalization and cardiovascular outcomes.

### Filling pressures analysis

In our study, sPAP and E/E`ratio, considered old surrogates for increased LVFP, demonstrated reduced diagnostic performance for HFpEF diagnosis, when compared to SR_CT and LAVimax. However, when included in a multivariate logistic regression, together with all the classical diastolic and strain parameters, only sPAP was incorporated in the model that best identified HFpEF, with p<0.001. Furthermore, we suggested new cut-off values to increase sensitivity for HFpEF diagnosis, for both sPAP (>33.5 mmHg) and E/E`ratio (>12), by comparison with values suggested by the scoring system.

E/E′ has been the main non-invasive surrogate parameter to predict LVFP. However, many studies demonstrated that E/E′ ratio has a weak correlation with pulmonary capillary wedge pressure (PCWP) in patients with HFpEF [43–48]. Overall, there is a lack of strong evidence in HFpEF patients supporting the utility of E/E′ as an accurate marker of LVFP. Even if the current guidelines [6, 10] use E/E’>15 as a parameter useful for HF diagnosis, this parameter demonstrated very low sensitivity in our study, in line with other studies. Horiuchi et al. found that only sPAP had a strong correlation with PCWP (R = 0.738, p<0.001), E/E`ratio having a weak correlation [49]. Another study demonstrated that tricuspid regurgitation velocity, used to non-invasively estimate sPAP, had the highest predictive value in identifying PCWP>15 mmHg (AUC = 0.89), compared to E/E’ and LAVimax [50]. The poor correlation of E/E’ ratio with LVFP in all studies might be explained by the fact that at least one third of patients with HFpEF may exhibit normal LVFP at rest, with elevated FP only on exertion [51]. In line with these findings, we also found a high performance for sPAP to identify HFpEF, when compared to E/E`ratio.

Lindqvist et al. [34], in a recent publication, demonstrated using invasive evaluation of the LA pressure, that SR_CT was the best accurate component of LA deformation measurements that correlated with PCWP. This parameter had superior accuracy in predicting elevated PCWP compared to recently proposed uni- and multivariable-based algorithms (r^2^ = 0.60, p < 0.001). Relationship was even stronger in patients with enlarged LA [34].

As 2019 scoring system suggested a multiparametric approach for HFpEF diagnosis, we also evaluated a combined parameter, composed by SR_CT, DI, sPAP, GS, LAVi min, these parameters exceeding the accuracy of conventional parameters. A combination of three from five parameters, was able to predict with the highest accuracy HFpEF diagnosis, by comparison with each parameter alone and by comparison with combined index from the scoring system. In these light, we suggest that these parameters should be validated in larger studies, in order to incorporate them in the scoring system, as functional and structural criteria. We also suggest that functional criteria might have higher contribution than structural ones, in a future alghorithm for HFpEF diagnosis, since than all functional parameters registered a higher accuracy than structural parameters. These parameters could be of particular interest to follow-up the preHFpEF patients to determine whether they progress to HFpEF.

### Limitations

Several limitations of our study should be acknowledged. The main limitation was the lack of invasive hemodynamic data. However, invasive assessment in our patients was not clinically indicated. Also, exercise stress echo was not performed in our study because we selected patients with a clear diagnosis of HFpEF. Another limitation was that it was a single centre and single vendor experience (GE). However, our subjects were rigorously selected, representing a reasonable sample. Finally, in terms ofapplicability in clinical practice, it is hard to be confident with strain rate values, since changes in the range of subunit are hard to manage. Most of HFpEF patients were in NYHA functional class II, with grade I and II of LVDD. Hence, further studies, with larger numbers of patients in NYHA functional class III or IV and grade III of LVDD are needed to validate the findings from our study.

## Conclusions

Our study demonstrated that by adding LA functional analysis, we might improve the HFpEF diagnosis accuracy, compared to present scoring system. Booster pump function is the only function that differentiates preHF from HFpEF patients. A value of SR_CT < -1.66/s outperformed conventional parameters from the scoring system, reservoir strain, and LA overload indices in HFpEF prediction. We also confirmed that none of the conventional parameters predict alone HFpEF diagnosis, making differentiation between HFpEF and preHF by echocardiography in resting condition impossible. The value of diagnosing HFpEF without the need for stress echo and invasive hemodynamic data is important, since not all patients are able to perform it in real world scenario. We suggest that functional LA analysis by STE could be used to improve diagnostic algorithms of HFpEF, and in the follow-up of HFpEF patients. Further studies are needed to validate our findings in a large population evaluated for HFpEF diagnosis.

## Supporting information

S1 File (DOCX)
