# Supplementary figures and images for: New insights into the potential utility of the left atrial function analysis in heart failure with preserved ejection fraction diagnosis
Source: PLoS One. 2022 May 4;17(5):e0267962. doi: 10.1371/journal.pone.0267962 (PMC9067684; doi:10.1371/journal.pone.0267962)

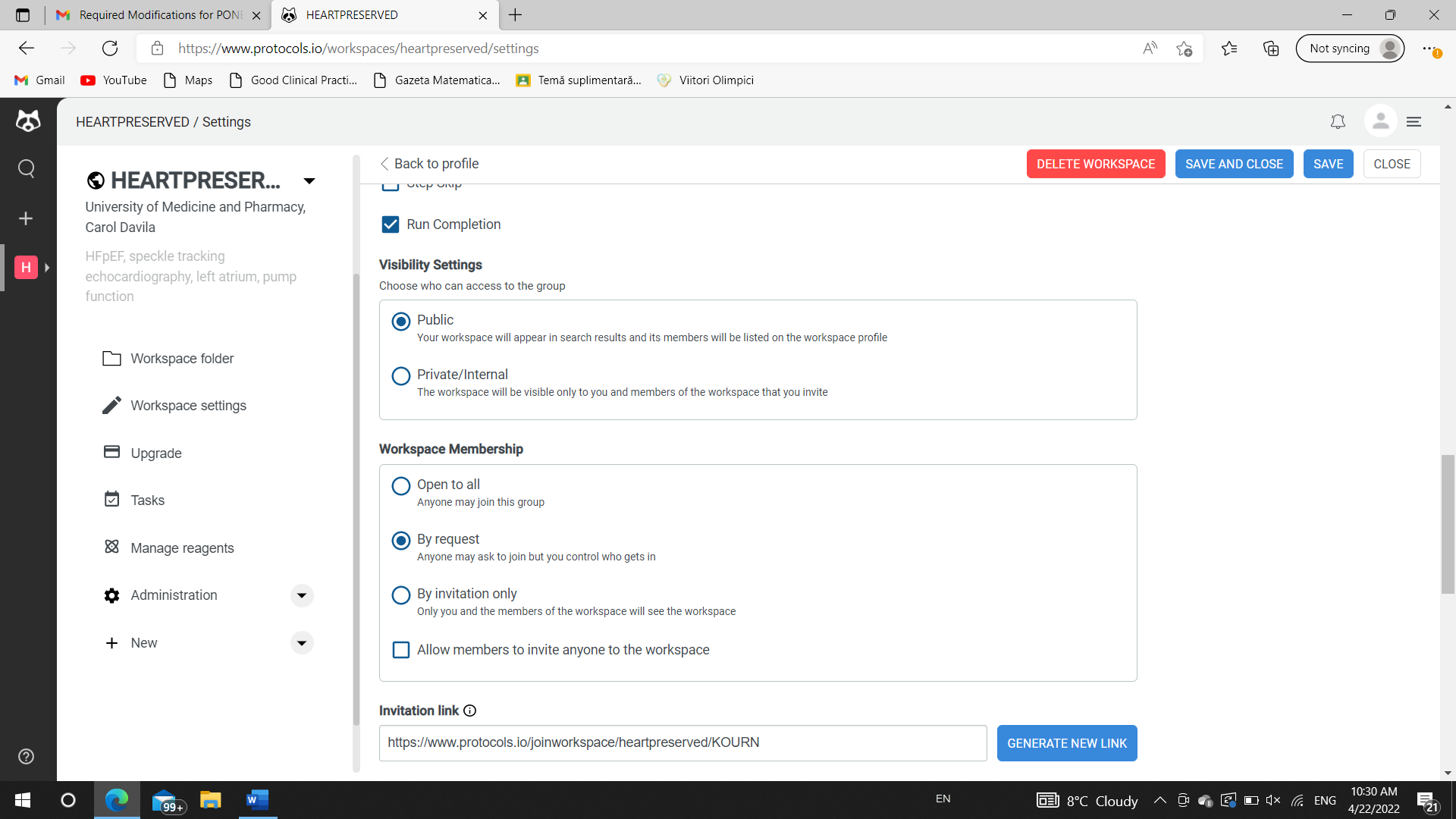

Supplement: S1 File — (DOCX) [file pone.0267962.s001.docx]
